# Teacher reports of emotional and behavioral problems in Nepali schoolchildren: to what extent do they agree with parent reports?

**DOI:** 10.1186/s12888-022-04215-4

**Published:** 2022-09-02

**Authors:** Jasmine Ma, Pashupati Mahat, Per Håkan Brøndbo, Bjørn H. Handegård, Siv Kvernmo, Anne Cecilie Javo

**Affiliations:** 1 Faculty of Health Sciences, Regional Centre for Child and Youth Mental Health and Child Welfare -North, UiT The Arctic University of Norway, Tromsø, Norway; 2 Child and Adolescent Psychiatry Unit, Kanti Children’s Hospital, Kathmandu, Nepal; 3 Centre for Mental Health and Counseling, Kathmandu, Nepal; 4 Department of Psychology, Faculty of Health Sciences, UiT The Arctic University of Norway, Tromsø, Norway; 5 Department of Clinical Medicine, Faculty of Health Sciences, UiT The Arctic University of Norway, Tromsø, Norway; 6 Sami National Competence Center for Mental Health (SANKS), Finnmark Hospital Trust, Sami Klinihkka, Karasjok, Norway

**Keywords:** Cross-informant, Teacher, Nepal, Children, Emotional and behavioral problems

## Abstract

**Background:**

Teacher reports of child emotional and behavioral problems (EBPs) are sparse in many low- and middle-income countries, especially when compared to reports from parents. Cross-informant information is pivotal to clinicians when dealing with mentally ill children. In this study from Nepal, we examined teacher reports of child EBPs, the agreement between teacher and parent reports, and how this agreement varied by type of EBP and child gender.

**Methods:**

This cross-sectional, observational study included 3808 schoolchildren aged 6–18 years from 16 districts of Nepal. Teacher and parent reports of EBPs were measured by the Nepali versions of the Teacher Report Form (TRF) and the Child Behavior Checklist (CBCL), respectively. Linear mixed model analysis was used for group comparisons and intraclass correlations. Agreement between TRF and CBCL scale scores were analyzed using Pearson’s correlation coefficient.

**Results:**

The prevalence of EBPs according to teacher reports was 15.4%, whereas the previous parent reported prevalence was 19.1%. Also, the mean TRF score was significantly lower than mean CBCL score for the 90 common items. Mean TRF scores for Total Problems, Externalizing Problems, and Internalizing Problems were 26.9 (standard deviation, SD 24.5), 6.1 (SD 7.2), and 7.9 (SD 7.3), respectively. Consistent with parent reports, mean TRF scores for Total Problems and Externalizing Problems were higher among boys than girls, whereas no significant gender differences were found for Internalizing Problems. Teacher-parent agreement was moderate (*r* = .38), and slightly higher for Externalizing Problems than for Internalizing Problems (*r* = .37 versus *r* = .34). Moderate to low correlations were found for all syndrome scales, with coefficients ranging from *r* = .26 (Social Problems) to *r* = .37 (Attention Problems). The effect of child gender on the teacher-parent agreement was significant for Internalizing Problems only, with a higher agreement for girls than for boys.

**Conclusion:**

Nepali teachers reported fewer child EBPs than parents. Teacher-parent agreement was moderate and varied by type of EBP and child gender. Our findings underscore the importance of obtaining information on child EBPs from both parents and teachers when evaluating and treating children in low- and middle-income countries like Nepal.

**Supplementary Information:**

The online version contains supplementary material available at 10.1186/s12888-022-04215-4.

## Background

Parents and teachers are common, important sources of information when assessing children’s emotional and behavioral problems (EBPs) [1]. Parents are important because they are familiar with the child’s behavior across many situations. Teachers are important because children spend a significant number of hours in school, thus teachers have ample time and opportunity to observe students’ behavior in a structured environment that is different from their home setting, and make comparisons among children of similar ages [2]. Because teachers can usually be reached relatively easily, their ratings of children’s behavioral problems are often used [3]. Although frequently used worldwide, teacher reports are still sparse in the low-and-middle-income countries (LMIC), and even more sparse when it comes to comparing teacher reports to parent reports. However, teachers’ observations are likely to vary based on the type of problems being rated (e.g., externalizing or internalizing problems) and the demographic characteristics of their students (e.g., gender, ethnicity, parental educational level) [4–6].

A meta-analysis by Rescorla and colleagues included studies from 21 countries that used the Teacher Report Form (TRF) to assess teacher reports of EBPs. It demonstrated that 15 of the 21 studies reported mean TRF scores for Total Problems within 1.0 standard deviation (SD, 6.2) of the overall mean of 21.6 [7] showing that despite differences across countries in their school systems, models of teaching, and perception of child problems, the mean TRF total scores were rather similar across many countries. The same meta-analysis showed that gender effects in teacher reports of EBPs were consistent across countries for Externalizing Problems and Attention Problems, with boys scoring significantly higher than girls in most countries. No large-scale studies on teacher reports of EBPs in Nepal have yet been published in the international literature [8]. Hence, the severity of child and adolescent EBPs as perceived in a school situation is still not known. Because children’s behavior is often situation-specific, the evaluation of their emotional and behavioral functioning in different social situations is an important and challenging part of clinical psychiatric assessment [9]. The gathering of information from multiple sources (e.g., teachers and parents), and settings (e.g., classroom and home) is considered best practice and is highly recommended to achieve a comprehensive picture [10–12]. Although the importance of using multiple sources of information when assessing child EBPs has been recognized worldwide, there has been little systematic research on teacher versus parent reports of child EBPs in LMICs. Studies from many countries have shown that differences in school structure (e.g., class size), parental involvement in school, as well as cultural differences in parent perceptions of child problems might impact the teacher-parent agreement on child EBPs [1]. However, the impact of such factors in LMICs might differ substantially from those in high-income-countries (HIC), and more studies from LMICs are warranted to explore potential differences and possible consequences.

Earlier studies have shown that parents tend to report more child EBPs than teachers. Studies of teacher and parent reports of EBPs, as measured by the TRF and the Child Behavior Checklist (CBCL), respectively, found that parents tended to report higher scores than teachers on all problem scales [13]. More recent studies comparing teachers’ and mothers’ ratings of the different types of EBPs arrived at similar conclusions [1, 14–16]. Studies from different countries have found low to moderate teacher-parent agreement on EBPs for the same child. A meta-analysis validity study from 2015, which included 341 studies worldwide, reported low to moderate cross-informant correspondence estimates (mean internalizing:* r* = 0.25; mean externalizing:* r* = 0.30; mean overall: *r* = 0.28) [17]. According to most studies, teacher-parent agreement was higher for externalizing problems than for internalizing problems [1, 18, 19]. This could be because externalizing problems are more visible and thus more likely to be noticed by both parents and teachers, resulting in more consistent ratings across different contexts [18, 20, 21].

Interestingly, results on the influence of child gender on teacher-parent agreement are inconsistent: some studies suggest that this agreement is not affected by child gender [22–24], while others have found that child gender does affect the agreement [1, 4, 14, 18, 25, 26]. Results on the impact of gender on cross-informant agreement also vary, with some studies reporting a higher teacher-parent agreement for girls [14, 18], and others reporting a higher agreement for boys [4, 25]. This inconsistency might be due to differences in the age groups studied, the use of different instruments, or comparisons of different problem scales. Cultural context might also affect cross-informant agreement for girls and boys [19].

At present, large-scale studies on teacher reports of EBPs are still sparse in low- and middle-income countries like Nepal [8]. Hence, the prevalence and magnitude of child and adolescent EBPs as perceived in a school situation is largely unknown. Moreover, no study has yet been performed in a Nepali cultural context on the effect of child gender on teacher-parent agreement. The aims of the present study were to examine the prevalence and magnitude of child EBPs in Nepal as reported by teachers, including different types of problems and possible gender differences. Further, to explore the agreement between teacher and parent reports, and how this agreement varied by type of EBP and child gender.

## Methods

This study presents information on teacher reports collected during a larger research project on the examination of EBPs of Nepali children from different castes and ethnic groups. The distribution of demographic data of the sample and the parent reports of EBPs have already been described in previous papers [27, 28].

### Participants and procedure

Within the framework of the research project, we purposively selected 16 districts from the three main ecological/geographical regions of Nepal, based on convenience and feasibility (three districts from the Mountain region, six districts each from the Middle Hills and the Tarai regions, and the Kathmandu district). We then purposively selected two governmental schools and two private schools from each district. Schools for children with special needs and faith-based schools were not included. Six students (three boys and three girls) from each grade level (grades 1–10) were then randomly selected using random number tables. Children who appeared in the school registration system, but were not attending the school, were not included. If schools did not have six children per grade, as was the case for some rural schools, we selected the remaining students needed from another, similar, nearby school in the same district. Thus, in each of the 16 districts, 240 children were selected, which gave a total of 3,840 children. The selection procedure has been reported in more details in previous papers [27, 28].

Twenty research assistants with a bachelor’s degree in education/psychology were responsible for data collection, supervised by seven field supervisors with a master’s degree in education or psychology and experience in data collection. Before commencing data collection, all research assistants and field supervisors attended an intensive, 3-day training program administered by the first author, during which attendees received instruction on the research project and instruments, their role and responsibilities, and thorough training in how to inform teachers and parents about the study, how to answer queries that might arise, and how to assist teachers and parents in completing the study forms. Throughout the data collection period, the work was monitored by the first author using frequent telephone check-ins, SKYPE meetings, and direct visits to the different districts.

After the schools were selected, research assistants met with and obtained written consent from school administrators. Research assistants and school administrators then held meetings with all teachers of students in grades 1–10 to inform them about the study. School administrators provided the parents of selected children with oral and written information and invited them to participate in the study. Teachers completed the Nepali version of the TRF for children aged 6–18 years (TRF/6–18) for the selected students in their class, and parents completed the Nepali version of the 2001 CBCL for children aged 6–18 years (CBCL/6–18). Research assistants collected data from the TRF and CBCL between September 2017 and January 2018. Data plotting was done manually during the first half of 2018 by three research assistants, supervised by the first author.

Out of 3840 selected students, 20 did not participate in the study, and 12 had missing information on the TRF. Thus 3808 students were included in the present analysis (99.2%).

### Measures

Both the TRF and the CBCL are included in the *Achenbach System of Empirically Based Assessment* (ASEBA) [29] and have been translated and adapted into the Nepali language by a Nepali researcher [30]. Both instruments have 118 specific problem items, which are scored on a three-point Likert scale (0 = absent, 1 = occurs sometimes, 2 = occurs often), plus two open-ended problem items. The TRF is based on the child’s functioning over the preceding 2 months, whereas the CBCL covers functioning over the preceding 6 months. Most of the items on the TRF have counterparts on the CBCL (90 common items, TRF_90_, CBCL_90_), but the CBCL items that teachers cannot assess (e.g., “have nightmares”) are replaced with items on behaviors they can observe (e.g., “disrupts class discipline”).

In both instruments, the problem items combine to form eight syndrome scales: Withdrawn/Depressed, Somatic Complaints, Anxious/Depressed, Rule-breaking Behavior, Aggressive Behavior, Social Problems, Attention Problems, and Thought Problems. There are some differences between the problem items that comprise the syndrome scales in the two instruments, the main one being in the Attention Problems scale, for which the TRF includes 26 items and the CBCL 10 items. Some of the syndrome scales are further condensed into two broad-band scales: Internalizing Problems (Withdrawn/Depressed, Somatic Complaints, and Anxious/Depressed) and Externalizing Problems (Rule-breaking Behavior and Aggressive Behavior). Finally, the Total Problems scale comprises all eight syndrome scales.

The internal consistency of the two instruments has been reported to be good across countries, with Cronbach’s alphas for the syndrome scales ranging from 0.72 to 0.95 on the TRF and from 0.72 to 0.94 on the CBCL [31]. Our previously published study showed that the alphas for the CBCL syndrome scales had overall good internal consistency [27]. The alphas for the TRF syndrome scales in the present study were: Anxious/Depressed: 0.80; Withdrawn/Depressed: 0.79; Somatic Complaints: 0.78; Social Problems: 0.74; Thought Problems: 0.74; Attention Problems: 0.91; Rule-Breaking Behavior: 0.74; and Aggressive Behavior: 0.89.

### Statistical analyses

SPSS statistics version 26.0 for Windows was used for all analyses. All CBCL information about prevalence and magnitude was taken from our previous paper [27]. To examine the prevalence rates of EBPs as reported by teachers, we used cut-off scores between the normal, borderline, and clinical groups based on American norms as described by Achenbach and Rescorla [31]. Since children are nested within grades and schools, linear mixed model (LMM) analysis was used for group comparisons of TRF scale scores. To measure the relative magnitude of the differences between means, i.e., the effect size, we calculated Cohen’s* d* [32]. Comparisons between genders on normal, borderline, and clinical status for the teacher data were computed using generalized LMM (GLMM; multinomial distribution, cumulative logit link function, random intercepts on both the class and the school level). Intraclass correlations (ICCs) of child EBPs among grades within schools (grade level) and among schools (school level) were computed using LMM via an unconditional means model [33]. ICCs are helpful to reveal dependency in the data among schools and grades within schools. A high ICC indicates high similarity between values from the same group. Comparisons of mean scores for the TRF_90_ and CBCL_90_ were analyzed using repeated-measures analysis of variance (rANOVA) [34]. For the rANOVA, we reported partial eta squared as an effect size measure. Partial eta square gives the proportion of the variance explained by a variable after accounting for other variables. In a model with just the informant (within-subject) variable, the partial eta squared is the proportion of the total variance explained by the informant variable.

Correlation between the TRF scale scores and the CBCL scale scores (teacher-parent agreement) was analyzed using Pearson’s correlation test. A Fisher Z-transformation was used to compare teacher-parent agreement between boys and girls. Here we applied the effect size measure q for guidance about the magnitude of the correlation difference [32]. In addition, we computed Q correlations as Spearman correlations for each child to assess the within-child association between teacher and parent scores on the TRF_90_ and the CBCL_90_, as recommended in the ASEBA manual [31]. The Q correlations are an alternative way of assessing cross-informant associations. Instead of testing the associations between scale scores for teachers, parents, and all participants combined, Q correlations use the TRF_90_ and CBCL_90_ to assess the associations of scores for each child. The Q correlation then shows how consistent the 90 items are, scored for a particular child. The significance level used for all tests was 0.005. We decided to use a low significance level because of the large sample size [35].

## Results

As in our previous study on parent reports of EBPs (reported using the CBCL) [27], in the present study on teacher reports, the majority of children had normal TRF scores for Total Problems, Internalizing Problems, and Externalizing Problems and this proportion was higher than for the CBCL. Prevalence of teacher reports of EBPs was 15.4%, compared to 19.1% for parent reports (Table 1). To examine the prevalence rates of EBPs, we used cut-off scores between the normal, borderline, and clinical groups based on American norms as described by Achenbach and Rescorla [31].

**Table 1:** Prevalence of emotional and behavioral problems for boys and girls as reported by teachers (parent reports to the right for comparison)

**TRF**	**Gender**			**CBCL** ^a^	**Gender**		
	Boys	Girls	Total		Boys	Girls	Total
Total Problems T score***				***Total Problems T score***			
Normal (< 60)	81.0%	71.1%	76.1%	Normal (< 60)	68.7%	71.5%	70.1%
Borderline (60–63)	6.6%	10.5%	8.5%	Borderline (60–63)	11.2%	10.4%	10.8%
Clinical (> 63)	12.4%	18.4%	15.4%	Clinical (> 63)	20.1%	18.1%	19.1%
***Internalizing problems T score***				***Internalizing problems T score*** ^*^			
Normal (< 60)	68.3%	66.8%	67.5%	Normal (< 60)	61.9%	66.9%	64.4%
Borderline (60–63)	8.0%	9.2%	8.6%	Borderline (60–63)	12.7%	10.3%	11.5%
Clinical (> 63)	23.7%	24.0%	23.9%	Clinical (> 63)	25.4%	22.8%	24.1%
Externalizing problems T score***				***Externalizing problems T score*** ^*^			
Normal (< 60)	78.0%	72.6%	75.3%	Normal (< 60)	76.5%	80.3%	78.4%
Borderline (60–63)	10.3%	10.1%	10.2%	Borderline (60–63)	7.9%	6.8%	7.4%
Clinical (> 63)	11.8%	17.3%	14.5%	Clinical (> 63)	15.6%	12.9%	14.2%

*TRF* Teacher Report Form, *CBCL* Child Behavior Checklist

^*^ *P* < 0.05; **P < 0.005; ***P < 0.0005

^a^ Data taken from reference [27]

### Intraclass correlations

The computations are based on a 3-level model where students (level1) are nested within grades (level 2) within schools (level 3). ICCs on the school level ranged from 0.10 to 0.16, indicating relatively large differences in problem means among schools. The ICCs on the grade level within schools were less than 0.02, and mostly smaller than 0.01. So the scores depended more on the school that children attended than on the grade they were in within the school (Table 2).

**Table 2:** Intraclass correlations (ICCs) for Teacher Report Form (TRF) scale scores by grade level and school level

TRF scales	ICC grade^a^	ICC school^b^
Total Problems	0.006	0.160
Externalizing	0.004	0.114
Internalizing	0.013	0.160
Aggressive Behavior	0.002	0.105
Rule-breaking Behavior	0.008	0.102
Attention Problems	0.003	0.124
Thought Problems	0.003	0.103
Social Problems	0.004	0.126
Somatic Complaints	0.013	0.159
Withdrawn/Depressed	0.004	0.111
Anxious/Depressed	0.019	0.147

^a^The proportion of the total variance among grades within schools

^b^The proportion of the total variance among schools

### The magnitude of teacher reports of emotional and behavioral problems for boys and girls

Because of high ICCs on the school level, we did a multilevel LMM analysis when comparing the genders. Significant differences between the genders were observed in mean TRF scores for all scales except Internalizing Problems, Somatic Complaints, and Withdrawn/Depressed. Boys had a significantly higher TRF score for the Total Problems scale than girls, mainly because of higher scores for the Externalizing Problems and Attention Problems scales. The effects of gender were mostly small; the largest effect was observed for the Rule-Breaking Behavior scale (standardized effect size: − 0.32) (Table 3).

**Table 3:** Mean overall and gender-specific Teacher Report Form (TRF) scale scores for Nepali schoolchildren

TRF scales	Gender				
	Boys (*N* = 1913) Mean^a^ (SD)	Girls (N = 1895) Mean (SD)	Total (IN = 3808) Mean (SD)	Gender effect F	Effect size^b^
Total Problems	29.09 (25.63)	24.60 (23.19)	26.85 (24.54)	37.87***	− 0.18
Externalizing Problems	7.13 (7.85)	5.15 (6.36)	6.14 (7.21)	82.02***	− 0.28
Internalizing Problems	7.70 (7.12)	8.18 (7.55)	7.94 (7.34)	4.70*	0.06
Aggressive Behavior	4.65 (5.50)	3.47 (4.67)	4.06 (5.13)	55.89***	− 0.23
Rule-Breaking Behavior	2.49 (2.81)	1.68 (2.11)	2.09 (2.52)	111.71***	− 0.32
Attention Problems	9.13 (8.12)	6.87 (7.10)	8.00 (7.71)	94.15***	− 0.30
Thought Problems	1.30 (2.05)	1.07 (1.81)	1.19 (1.94)	13.89***	− 0.12
Social Problems	2.62 (2.79)	2.28 (2.58)	2.45 (2.70)	17.05***	− 0.13
Somatic Complaints	1.32 (2.10)	1.48 (2.22)	1.40 (2.16)	5.86*	0.07
Withdrawn/Depressed	2.32 (2.60)	2.31 (2.64)	2.32 (2.62)	0.03	− 0.01
Anxious/Depressed	4.07 (3.76)	4.40 (4.06)	4.23 (3.91)	8.01**	0.08

*SD*: Standard deviation

^*^ *P* < 0.05; **P < 0.005; ***P < 0.0005

a. The table shows estimated marginal means

b. Negative effect size means higher scores for boys

### Comparison of mean scores for the 90 common items on the Teacher Report Form and Child Behavior Checklist

A repeated-measures ANOVA showed that the mean TRF_90_ score was significantly lower than the mean CBCL_90_ score. The partial eta squared of 0.048 gives the effect of the difference between the informants (Table 4).

**Table 4:** Comparison of mean scores for the 90 common items on the TRF and CBCL (TRF_90_ and CBCL_90_)

TRF_90_ score Mean (SD)	CBCL_90_ score Mean (SD)	F	Partial Eta Squared
19.25 (17.76)	24.08 (20.49)	190.70***	0.048

^***^ *P* < 0.0005. F: within-subject effect

### Teacher-parent agreement for scale scores and the effect of gender

Moderately positive and significant agreement was found between all TRF and CBCL problem scales. The largest cross-informant *r*s were for Attention Problems (*r* = 0.37) and Externalizing Problems (*r* = 0.37). We found a significant gender effect only for Internalizing Problems (z = –2.87; p = 0.004), with a higher agreement for girls than for boys. No gender effects were found for any of the syndrome scales. All differences in agreement between genders were small with effect sizes q < 0.10 (Table 5).

**Table 5:** Teacher-parent agreement and the effect of gender

Scales	Pearson’s correlation	Correlation for boys	Correlation for girls	Z test ^Ω^	Effect size q^a^
Total Problems	0.38 **	0.36	0.40	–1.16	0.04
Externalizing Problems	0.37 **	0.37	0.35	0.83	0.03
Internalizing Problems	0.34 **	0.30	0.38	–2.87**	0.09
Aggressive behavior	0.33 **	0.33	0.31	0.65	0.02
Rule-Breaking behavior	0.36 **	0.37	0.30	2.45	0.08
Attention Problems	0.37 **	0.35	0.39	–1.52	0.05
Thought Problems	0.29 **	0.27	0.31	–1.24	0.04
Social Problems	0.26 **	0.25	0.26	–0.40	0.01
Somatic Problems	0.33 **	0.31	0.36	–1.49	0.05
Withdrawn/ Depressed	0.28 **	0.25	0.32	–2.22	0.07
Anxious/ Depressed	0.28 **	0.24	0.31	–2.44	0.08

^*^ *P* < 0.05; **P < 0.005; ***P < 0.0005

^Ω^ The Z is a test statistic used to test for gender differences for the correlations; a. effect size q =|z_1_ – z_2_|, where z_i_ = .5*ln((1 + r_i_)/(1-r_i_)), and r_1_ represent the correlation for boys and r_2_ represent the correlation for girls

**Fig. 1:**
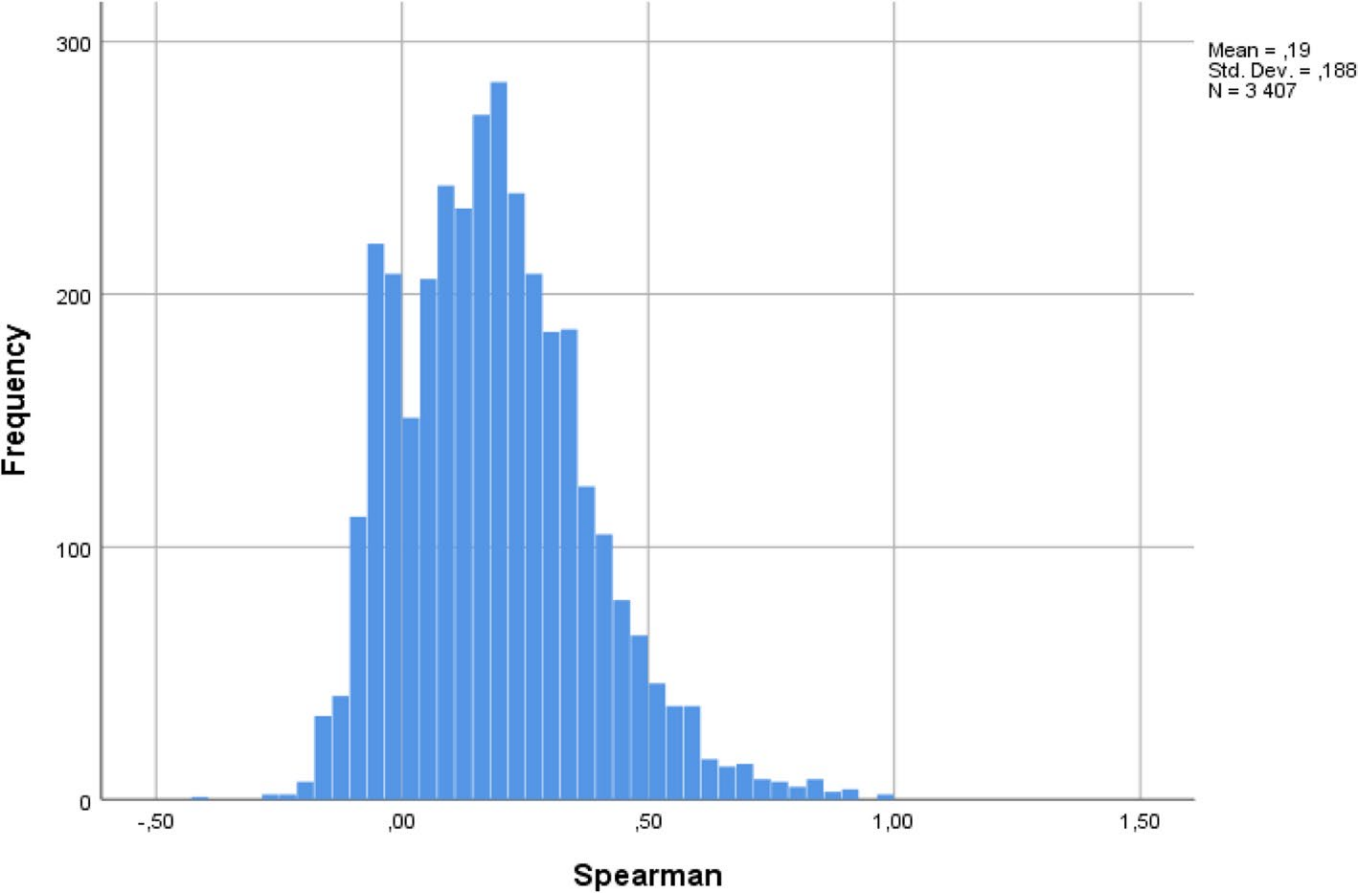
Within-child association between teacher and parent scores on the TRF_90_ and the CBCL_90_ (Spearman correlations for each child)

In the 2001 ASEBA manual, the mean Q correlation for comparing individual CBCL and TRF data is given as 0.23 [30]. We found a mean correlation of 0.19, which is slightly below this reference sample mean. The mean Q correlation of 0.19 indicates low agreement in ratings (Fig. 1).

## Discussion

This study assessed the prevalence and magnitude of teacher reports of EBPs among schoolchildren in Nepal and is the first study to investigate teacher-parent agreement on child EBPs in Nepal. The prevalence of EBPs reported by teachers was found to be 15.4% which is lower than the previous parent reported prevalence of 19.1% [27]. Also, the mean TRF_90_ score of Total Problems was lower than the CBCL _90_ score which is consistent with findings from other international studies [1, 14]. Similar to the previously reported prevalence by the parents [27], teachers reported a higher prevalence of internalizing problems than externalizing problems (23.9% versus 14.5%). We do not know the reason for the higher internalizing problems in our study and more studies are warranted to explore possible cultural reasons.

Based on data from 21 societies, Achenbach and Rescorla constructed different norm groups (high, medium, and low) for the TRF. When they averaged the TRF scores for Total Problems, they observed an omni-cultural mean of 21.6 (SD 6.2) [7]. Nepal has not yet been ranked based on these norm groups due to the lack of internationally published scientific studies. However, the TRF mean score for Total Problems of 26.9 (SD 24.5) that we observed suggests that Nepal should be placed within the group of medium-scoring countries.

Similar to findings from the meta-analyses by Rescorla et al. [1, 7], the present study showed that teacher reported scores for Total Problems and Externalizing Problems were significantly higher for boys than girls. Contrary to the Rescorla et al. studies, gender differences were not significant for Internalizing Problems. Girls had more internalizing problems than boys in most countries, but we saw no gender difference in this study. However, gender differences in teacher reports of EBPs were in line with those seen in the parent reports in our previous study of the same sample [27].

Our study showed that the teacher-parent agreement on scale scores for the same child was low to moderate, which is in line with most other studies [1, 3, 14]. The discrepancy between teacher and parent reports might be due to different observation contexts. Indeed, children’s behavior may vary in the home and at school, which may give rise to a lack of consistency in many cases [3]. Another possible explanation for the discrepancy in reports might be that parents and teachers have different emotional relationships with children and different expectations of their behavior [36]. Equally important, as noted by De Los Reyes, parents and teachers may have different “decision thresholds” for considering a child’s behavior as problematic or deviant [10]. As suggested by some studies, one reason for the lower frequency of teacher reports of EBPs may be that teachers are more familiar with age-appropriate behaviors, and therefore more tolerant towards certain behavioral problems than parents [3, 4]. Lower ratings might also be due to the fact that teachers look after a larger number of children, which may make it difficult for them to discern individual children’s problems. With large class sizes, teachers cannot be fully aware of their students’ behavior, which might affect their ratings, and consequently, teacher-parent agreement [21]. In their large-scale study of children from 21 countries, Rescorla and colleagues found that large class size was the one characteristic most associated with lower levels of parent-teacher agreement. Children from the largest classes (i.e., 40 children) tended to have a low parent-teacher agreement (r ≤ 0.20), whereas those from the smallest class sizes (15–25 students) had the highest agreement (*r* = 0.49) [1]. Many governmental schools in Nepal have large class sizes: up to 40 students per class. An additional explanation could be that the level of contact between parents and teachers in Nepal might be rather low, especially in rural areas. This might limit parents’ and teachers’ possibilities to share information about the child, which again might lead to lower levels of agreement on child EBP. Other studies have found that limited contact and shared information are associated with lower teacher-parent agreement [1]. However, the hypotheses mentioned above were not examined in the present study. Future research is warranted to explore in more detail the different mechanisms which may underlie cross-informant discrepancies in ratings in a Nepali context.

Consistent with previous studies [1, 4, 18, 21], our study suggested that the teacher-parent agreement was higher for externalizing than for internalizing problems. One explanation for this may be that internalizing problems are difficult for teachers to recognize, and that withdrawn/depressed behavior and anxiety are more likely to be observed by the parents. This argument might also be valid for our Nepali study. However, more detailed studies are needed to verify the hypothesis.

The highest teacher-parent agreement for the syndrome scales was found for Attention Problems. As suggested by other studies, attention problems in children appear to be more stable across various contexts, such as home and school [18], which may cause higher teacher-parent agreement. However, there might be other reasons as well. In Nepal, parents strongly emphasize the importance of children’s academic achievements in school and tend to regard attention problems as linked to academic difficulties. Thus, the frequency of contact between parents and teachers regarding attention problems might be higher than for other problems. Frequent communication between parents and teachers may, in turn, lead to a common understanding of problems, and subsequently, to a higher cross-informant agreement. An interesting topic in future Nepali studies might be to examine the frequency and content of the contact between parents and teachers on children’s attention problems to see if such factors might impact the cross-informant agreement.

In the present study, we found a significant gender effect on teacher-parent agreement for internalizing problems, with an agreement that was higher for girls than boys. This finding is consistent with other international studies [1]. No gender effect was found for externalizing problems, which differs from many international studies that have reported a higher agreement for boys than for girls [1, 4, 21]. Different results across countries suggest the need to further examine child gender as a moderator of cross-informant agreement. We do not know why teachers and parents in Nepal agreed more on girls’ internalizing problems. It is possible that girls with anxiety/depressed problems might display more consistent behaviors across different environments, leading parents and teachers to agree more on such symptoms. It may also be that teacher–child conflicts increase the discrepancy between problems reported by teachers and parents, and that teachers experience less conflicts with girls than boys. In Nepal, girls are subject to more control, are less likely to communicate their distress, and tend to behave in a more submissive manner than boys [37], which might create less conflicts with teachers. However, more Nepali studies are warranted to confirm this hypothesis and to examine other cultural factors that might account for variations in teacher-parent agreement.

### Limitations of the study

This study has its limitations. One limitation is that we used the American norms as cut-offs for the TRF and CBCL, as Nepali norms for these instruments are still lacking. Without Nepali norms, the reported differences in the prevalence of externalizing and internalizing problems, and in girls and boys, may be inaccurate. Moreover, although the selection of children in each school was random, the purposive selection of districts and schools could have been a source of selection bias. Hence, we cannot claim that the results are representative of the whole country. Additional research on teacher-parent agreement in clinical samples in Nepal is needed to test the generalizability of our findings. Another limitation of this study is that data was collected from teachers and parents only, and not from the children themselves. It is widely acknowledged that children are the key informants and experts on their own lives, and their opinion should be asked when assessing their mental health needs [38]. Youths’ self-reports might have broadened our understanding and identified more children who struggle with their emotions or behaviors. Finally, our data was informant data. Additional observational data would have granted us more certainty in determining whether the teacher-parent discrepancies reflected true differences in child behavior between school and home. It should be noted that the scope of this study did not include the examination of other socio-cultural or family factors that might have impacted teacher-parent agreement on child EBP.

## Conclusion

The prevalence and magnitude of teacher reports of EBPs in Nepali children were similar to those found in other parts of the world. We found a lower level of EBPs compared to parent reports, and moderate parent-teacher agreement, which is in line with most international studies.

In a clinical setting, it is important to obtain information from different sources, such as teachers and parents, to systematically assess child problems. The present study provides more knowledge on teacher reports of child EBPs in Nepal and shows how child EBPs might vary in the school and home contexts. Hopefully, our findings will inspire clinicians to include different sources of information when assessing children admitted to mental health care services. This study may also be used as a springboard for future studies on the contextual factors that impact child EBPs in Nepal.

## Supplementary Information

**Additional file 1.**

## Data Availability

The datasets used and /or analysed during the current study are included in the supplementary information file.

## Abbreviations

EBP: Emotional and behavioral problems
TRF: Teacher Report Form
CBCL: Child Behavior Checklist
SD: Standard deviation
ASEBA: Achenbach System of Empirically Based Assessment
LMM: Linear mixed model
GLMM: Generalized linear mixed model
ICC: Intraclass correlations
ANOVA: Analysis of variance
CWIN: Child Workers in Nepal
FORUT: Solidarity Action for Development, Norway FORUT
